# Refugee, Migrant and Asylum Seekers’ Experience of Accessing and Receiving Primary Healthcare in a UK City of Sanctuary

**DOI:** 10.1007/s10903-021-01227-2

**Published:** 2021-06-17

**Authors:** Rosie Scott, Emer Forde, Clare Wedderburn

**Affiliations:** GP Centre, Bournemouth University, Gateway Building, St Paul’s Lane, Bournemouth, BH8 8GR UK

**Keywords:** Primary care, General Practice, Access to healthcare, Education

## Abstract

The World Health Organisation estimate there are about 1 billion migrants in the world today. The scale of population movement and a global refugee crisis presents an enormous challenge for healthcare provision, and too often the specific health needs of refugees and migrants are not met. This study assessed refugee, asylum seeker and vulnerable migrants’ (AMRs') experience of front line primary healthcare in a region of the United Kingdom designated as a ‘City of Sanctuary’. A questionnaire study explored the views of people seeking refuge and third sector workers supporting them. The majority of AMRs were registered with a GP and positive about their consultations. The views of third sector workers provided a less favourable window into their experience of primary care. In conclusion, the work highlighted patchy experience of primary care, even in a region of the UK designated as a ‘City of Sanctuary’ for people seeking refuge. There is a need for further education of rights to care in the UK, information for people on how to navigate local healthcare systems, consistent access to routine health checks and translation services.

## Background and Theoretical Framework

According the World Health Organisation (WHO), there are an estimated 1 billion migrants in the world, of whom 258 million are international migrants. Drivers leading to large scale population movement include climate change, conflict, political oppression and desire for greater economic opportunity. Although COVID-19 has disrupted all forms of human mobility, many of these drivers are likely to increase in the future and the needs of asylum seekers, migrants and refugees (AMRs) must be embedded in all healthcare systems.

AMRs’ health needs are often complex and previous literature has identified barriers to accessing health services such as inadequate information on how to navigate healthcare systems in host countries, language, stigma, and poor continuity of care [1–6]. General Practitioners (GPs) provide front line healthcare in the UK and are the gateway to all other specialist services. A study on AMRs’ experiences of accessing general practice over a decade ago identified a need for professional interpreters; information on how to access healthcare; and education for GPs on refugee health [4]. The current study provides an up-to-date exploration of whether these suggestions have been implemented during the intervening years. Our focus of interest was in a region designated as a UK ‘City of Sanctuary’ where healthcare systems should be equipped to provide high quality care for AMRs [7]. We explored the views of AMRs and third sector workers (TSW) supporting them. Previously, we reported on local doctors’ knowledge of AMR health [8]. This triangulation helps identify gaps and improves our understanding of whether healthcare infrastructures are meeting the needs of the local population.

### Research Questions

1. Are AMRs able to access primary care?
2. Do they understand their rights to free primary care and how to navigate local healthcare systems?
3. What is their experience of primary healthcare in a ‘City of Sanctuary’?
4. Do TSWs provide an alternative view?

## Method

22 adult AMRs and 10 TSWs were recruited through The Red Cross and International Care Network (ICN), who together supported 483 adult AMRs. The charities were accessed through contracted work from the council, other local agencies and word of mouth. Bournemouth, Christchurch and Poole is not only designated as a ‘City of Sanctuary’ but also home to unaccompanied young refugees and has resettled 8 families (16 adults) as part of the Syrian Vulnerable Person Resettlement Scheme. The council serves a general population of 187,503 people.

In the AMR group, there were 18 females, 2 males and 2 did not answer. Countries of origin included Iran, Afghanistan, Turkey, Portugal, China, Brazil, Korea, Albania, Iraq, Eritrea, and Sudan. Age range 18–69 years. Time in the UK varied from less than 1 year to 6 years. Demographic data for TSWs was not collected. Participants were given an information sheet and author RS attended drop-in sessions to provide information and answer questions. All questionnaires were anonymous. Some participants completed these alone and others with help from a relative or TSW. Early drafts of the questionnaire were screened by TSWs to help reduce medical jargon. Questions were typically ‘yes/no/not sure’ or 5 point Likert scales. For example, rating experience from ‘very good’ to ‘very bad’. Likert scales also included thumbs up/down/neutral and smiling/frowning/neutral faces to help improve communication. Ethics approval was obtained from Bournemouth University’s Science, Technology and Health Research Ethics committee.

## Results

### Access to Primary Care

AMR: 95% were registered with a GP. 5% had been refused registration and did not know why. 36% had a general health check. Of those who had not, 82% would like one. 74% eligible had cervical screening and 100% mammograms, as part of routine NHS programmes.

TSW: 70% reported difficulties in registering clients with a GP. The reasons included surgeries asking for proof of ID/address when none was available (40%), language barriers (20%) and practices not being aware of entitlements to care (20%).

### Understanding Rights to Care and the Local Healthcare System

AMR: 95% knew that they had a right to free primary care. Only 36% knew how to access a GP out of hours. 41% had called 999 (UK emergency service for acute serious problems) and 36% had been to Accident & Emergency, higher than the general population.

TSW: Only 30% felt confident in their knowledge of AMRs’ rights to care. Only 10% were correct in who can access free primary care. 90% felt that AMRs use of GP services was ‘frequent/very frequent’ compared to the general population and 50% rated use of A&E as ‘frequent/very frequent’. One TSW commented: “I know people who have given up on their GPs and who fail to seek care even when they really need it. This has sometimes ended in needing to go to A&E.”

### Experience of Primary Care

AMR: 79% reported their GP experience as ‘good/very good’. Regarding ‘what was good?’ AMRs commented on GPs being friendly and kind with good listening skills. On ‘what could have been better?’ comments included doctors having a greater awareness of other cultures, giving medicine, access to interpreting services and with the doctor being more patient. 32% agreed with the statement “The GP had problems understanding me”, 18% felt “the GP did not understand my cultural background” and 32% did not “fully understand the advice or treatment given”.

68% AMRs reported needing someone to help translate and 82% of the time this was a relative. Only 27% AMRs reported a professional translator being available.

36% AMRs felt their health was better than when they had arrived in the UK but 36% felt it was worse. 23% reported having longterm physical illnesses and 60% felt they were receiving help. 23% reported having mental health problems and only 40% reported receiving help.

TSW: 60% felt that AMRs’ health needs were met in GP consultations most or all of the time. Physical (60%) and social needs (60%) were more likely to be met than mental health needs (10%). Regarding ‘what was good?, TSWs reported that GP staff were generally helpful and welcoming, some gave extra time and spoke slowly. However, many aspects could have been better: doctors addressing the client rather than TSW; access to translators; more time. TSWs were specifically asked about a range of challenges highlighted in previous literature (see Table 1).

**Table 1:** Do you think any of the following challenges are ones that refugee/migrants experience in consultations with GPs?

	% Third sector workers who said “yes”
Language barriers	100
Different expectations	100
Client did not understand the GPs explanation of the treatment plan	90
Lack of interpreting services	90
Cultural differences/lack of understanding of backgrounds	90
Consultation time was too short	80
Poor continuity of care	50
Inappropriate/unacceptable treatment/outcome	40
Racial prejudice	30

90% of TSW said an interpreter was required and in only 10% cases a professional translator was available. 100% felt that GPs needed more education and AMRs needed more information about how the NHS works, as well as professional translators. 90% agreed it would be useful to have a local GP with specialist knowledge of refugee health. Other suggestions included: female doctors for female patients; women’s health clinics, antenatal classes, improved access to mental health services; better understanding, diagnosis and treatment on the first visit rather than “brushing them off with a prescription”; specialist referrals before a problem deteriorated; multilingual doctors.

## Discussion

The majority of AMRs were registered with a GP and linked into NHS screening programmes. They were generally positive about GP consultations, particularly the personal characteristics of doctors being kind and showing good listening skills. This is important as trust and respect are key facilitators to good health [5]. One limitation of our report is the relatively small sample size, although comparable with other studies [4, 5], and similar levels of registration were reported in a recent policy report in Wales [2]. Interestingly, 79% AMRs in Wales had an initial health assessment, as recommended by the WHO and the UK government [9]. However, only 36% AMRs had in our study, suggesting this provision is patchy across the UK.

All studies recruiting participants through charitable organisations may have a positive bias, as people not accessing support could be even more marginalized and face greater challenges accessing healthcare [6]. For this reason, we sought an alternative window into AMR health through TSWs. They reported difficulties registering patients, as well as specific challenges such as language barriers and differing expectations. Some TSWs and local doctors [8], were not confident in AMRs entitlements to NHS care. In response, we have introduced Doctors of the World ‘Safe Surgeries’ education [10] for all GP trainees in our region. Author RS ran a session on how to navigate the healthcare system for local AMRs. Our report highlights that people seeking sanctuary still need information on local healthcare systems, consistent access to routine health checks, professional translators and mental health services. Further work is needed to measure and improve AMRs access to primary care, particularly in places designated as ‘Cities of Sanctuary’.
